# A role of the adaptive immune system in glucose homeostasis

**DOI:** 10.1136/bmjdrc-2015-000136

**Published:** 2016-02-15

**Authors:** Laura L Bronsart, Christopher H Contag

**Affiliations:** 1 Department of Biology, Stanford University, Stanford, California, USA; 2 Departments of Pediatrics, Radiology, Microbiology & Immunology, Stanford University, Stanford, California, USA

**Keywords:** Glucose Tolerance, Insulin Resistance, Inflammation, Type 2 Diabetes

## Abstract

**Objective:**

The immune system, including the adaptive immune response, has recently been recognized as having a significant role in diet-induced insulin resistance. In this study, we aimed to determine if the adaptive immune system also functions in maintaining physiological glucose homeostasis in the absence of diet-induced disease.

**Research design and methods:**

SCID mice and immunocompetent control animals were phenotypically assessed for variations in metabolic parameters and cytokine profiles. Additionally, the glucose tolerance of SCID and immunocompetent control animals was assessed following introduction of a high-fat diet.

**Results:**

SCID mice on a normal chow diet were significantly insulin resistant relative to control animals despite having less fat mass. This was associated with a significant increase in the innate immunity-stimulating cytokines granulocyte colony-stimulating factor, monocyte chemoattractant protein 1 (MCP1), and MCP3. Additionally, the SCID mouse phenotype was exacerbated in response to a high-fat diet as evidenced by the further significant progression of glucose intolerance.

**Conclusions:**

These results support the notion that the adaptive immune system plays a fundamental biological role in glucose homeostasis, and that the absence of functional B and T cells results in disruption in the concentrations of various cytokines associated with macrophage proliferation and recruitment. Additionally, the absence of functional B and T cells is not protective against diet-induced pathology.

**Key messages**

- Mice lacking of an adaptive immune system (SCID mice) have disruptions in the concentrations of various circulating cytokines.
- SCID mice have significant glucose intolerance that is independent of diet and obesity.
- The absence of functional B and T cells in SCID mice is not protective against diet-induced glucose intolerance progression.

## Introduction

Increasing interest in the convergent biology of insulin resistance and diabetes mellitus has been partly fueled by the need for effective interventions, and by recent data implicating the immune system in the pathogenesis of these diseases and perhaps shared mechanisms. It has long been established that obesity, insulin resistance, and chronic inflammation are often associated, but it has been challenging to determine if a causal relationship exists among these conditions.

It is known that the adipose tissue of obese mice and humans is characterized by an accumulation of macrophages and, it is thought, that this is a necessary component of disease progression.^1–3^ The secretion of cytokines tumor necrosis factor-α and interleukin (IL)-6 by infiltrating macrophages are thought to play a direct role in reducing the insulin sensitivity of regional adipocytes.^4–6^ Even more compelling, the role of the immune system in insulin resistance is no longer thought to be restricted to just the innate arm, but also includes an adaptive immune response. B and T cells have both been implicated in insulin resistance, with both detrimental and protective effects being reported.^7–21^

The results to date have been compelling; however, they fail to address if the adaptive immune system is a necessary component for metabolic homeostasis; that is, by manipulating the immune phenotype are we disrupting a physiological homeostatic mechanism? This study aimed to examine this by assessing the effects of immune incompetence on metabolic homeostasis in the presence and absence of a high-fat diet (HFD).

## Research design and methods

### Ethics statement

All animal studies were completed in accordance with the Guide for the Care and Use of Laboratory Animals of the National Institutes of Health and approved by the Institutional Animal Care and Use Committee of Stanford University.

### Mouse manipulations

Male, 6-week-old BALB/c control and BALB/c *scid* mice were purchased from Jackson Laboratories. Blood glucose measurements were taken using the One Touch Ultra blood glucose meter. The HFD, 60% kilocalories from fat, (#D12492) and control, 10% kilocalories from fat, (#D12450B) diets were purchased from Research Diets, Inc. Fasting insulin levels were detected using the Crystal Chem Inc Ultra Sensitive Mouse Insulin ELISA (#90080). The Stanford Human Immune Monitoring Core completed the mouse cytokine Luminex array. The Stanford Department of Comparative Medicine's Histology Lab prepared all histological samples.

### Glucose tolerance test

The glucose tolerance test (GTT) was completed as previously described.^22^ Briefly, mice were fasted for 8 h followed by intraperitoneal administration of 2 g/kg of D-glucose.

### Insulin tolerance test

The insulin tolerance test (ITT) was completed as previously described.^22^ Briefly, mice were fasted for 6 h followed by intraperitoneal administration of 2 units/kg of Humulin R insulin.

### Statistical analysis

All data were presented as the mean±SEM. For data analysis between two groups, significance was determined by the unpaired Student t test and defined as p≤0.05. For repeated measures data (GTT and ITT), significance was determined by two-way analysis of variance for repeated measures with Bonferroni correction and defined as p≤0.05.

## Results

**Figure 1:**
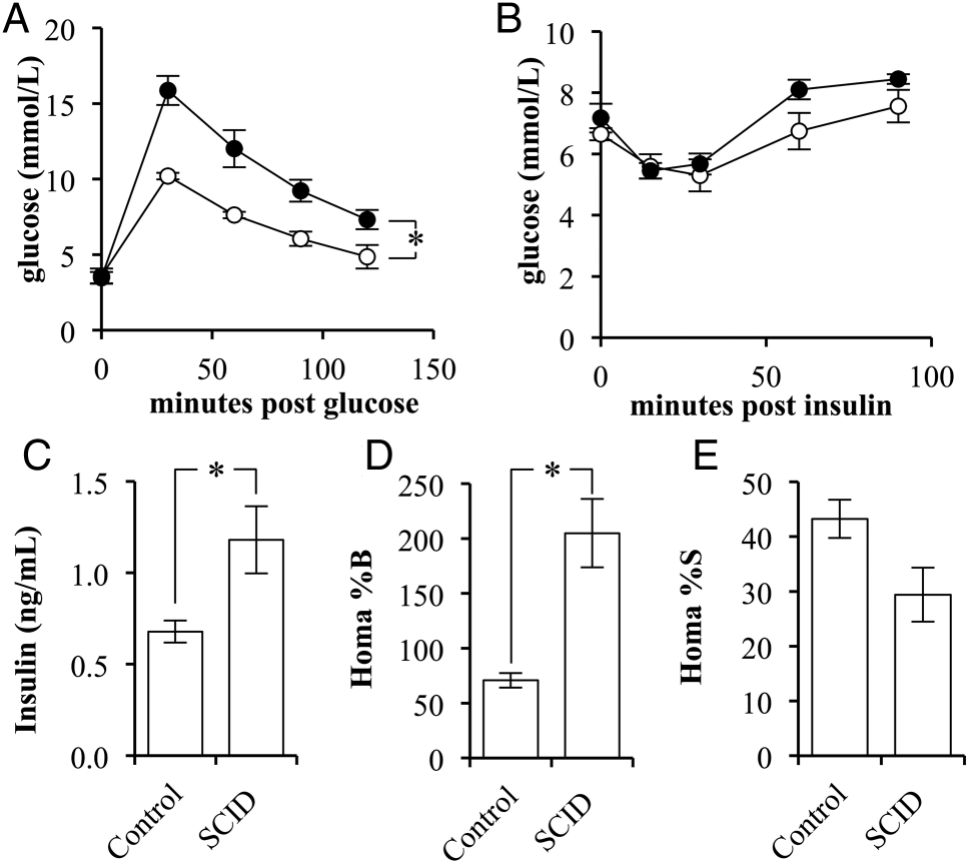
The insulin sensitivity of BALB/c *scid* mice is lower than that in BALB/c mice. (A) Glucose tolerance test of control (white circles) and SCID (black circles) mice following a 2 g/kg glucose challenge (n=4/5). (B) Insulin tolerance test of control (white circles) and SCID (black circles) mice following a 2 units/kg insulin challenge (n=4/5). (C) The fasting, blood-insulin levels of control and SCID mice (n=4/5). (D) The per cent β-cell function as determined by the homeostatic model assessment (HOMA) of control and SCID mice (n=4/5). (E) The per cent insulin sensitivity as determined by the HOMA of control and SCID mice (n=4/5). (Error bars represent the mean±SEM, and *signifies a significant difference between the designated groups of p<0.05.)

The phenotypes of BALB/c *scid* (SCID) and the BALB/c strain controls (control) were compared for variations in glucose and insulin sensitivity. SCID mice had significantly reduced glucose tolerance compared with control mice (p=0.0049; figure 1A). There was no difference observed between the fasting blood glucose levels or in response to insulin administration (figure 1B).

On evaluation of fasting insulin levels, SCID mice had a 174% greater fasting blood insulin concentration than that of controls (p=0.0345; figure 1C). Use of the homeostatic model assessment method indicated that SCID mice had significantly greater β-cell function, 205±31%, when compared with control mice, 71±7% (p=0.0183; figure 1D). Correspondingly, the method indicated that SCID mice had a nearly significant reduction in insulin sensitivity (p=0.0977; figure 1E).

**Figure 2:**
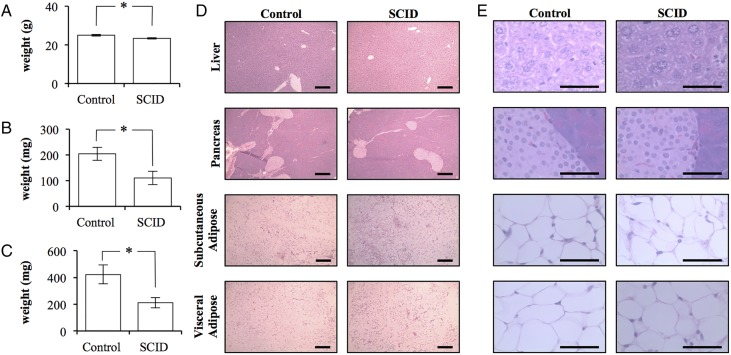
The adipose tissue of BALB/c *scid* mice is quantitatively, but not qualitatively, different than that of BALB/c control mice. (A) The total body weight of control BALB/c and BALB/c *scid* (SCID) mice (n=4/5). (B) The total weight of the subcutaneous adipose tissue bilaterally isolated from the inguinal region of control and SCID mice (n=4/5). (C) The total weight of the epididymal adipose tissue bilaterally isolated from control and SCID mice (n=4/5). (Error bars represent the mean±SEM, and *signifies a significant difference between the control and SCID groups of p<0.05.) (D) 10× light microscopy of H&E stained sections of liver, pancreas, subcutaneous, and visceral adipose tissue collected from control and SCID mice. Scale bar is 200 μm. (E) 100× light microscopy of H&E stained sections of liver, pancreas, subcutaneous, and visceral adipose tissue collected from control and SCID mice. (Scale bar is 50 μm.)

Despite the apparent insulin resistance of SCID mice, SCID mice weighed significantly less at 23.3±0.3 g (p=0.0332; figure 2A). The total body weight of control mice was 25.0±0.4 g. This difference was predominately due to differences in fat mass. SCID mice had 50% the adipose volume when compared with control mice. SCID mice had 110±26 mg of inguinal subcutaneous adipose tissue and 211±37 mg of visceral epididymal adipose tissue (mean±SEM). In contrast, control mice possessed significantly more adipose tissue with 204±25 and 423±70 mg, respectively (p=0.0383 and 0.0252; figure 2B,C). Histopathology did not reveal any clear differences between the tissues collected from mice (figure 2D,E).

**Figure 3:**
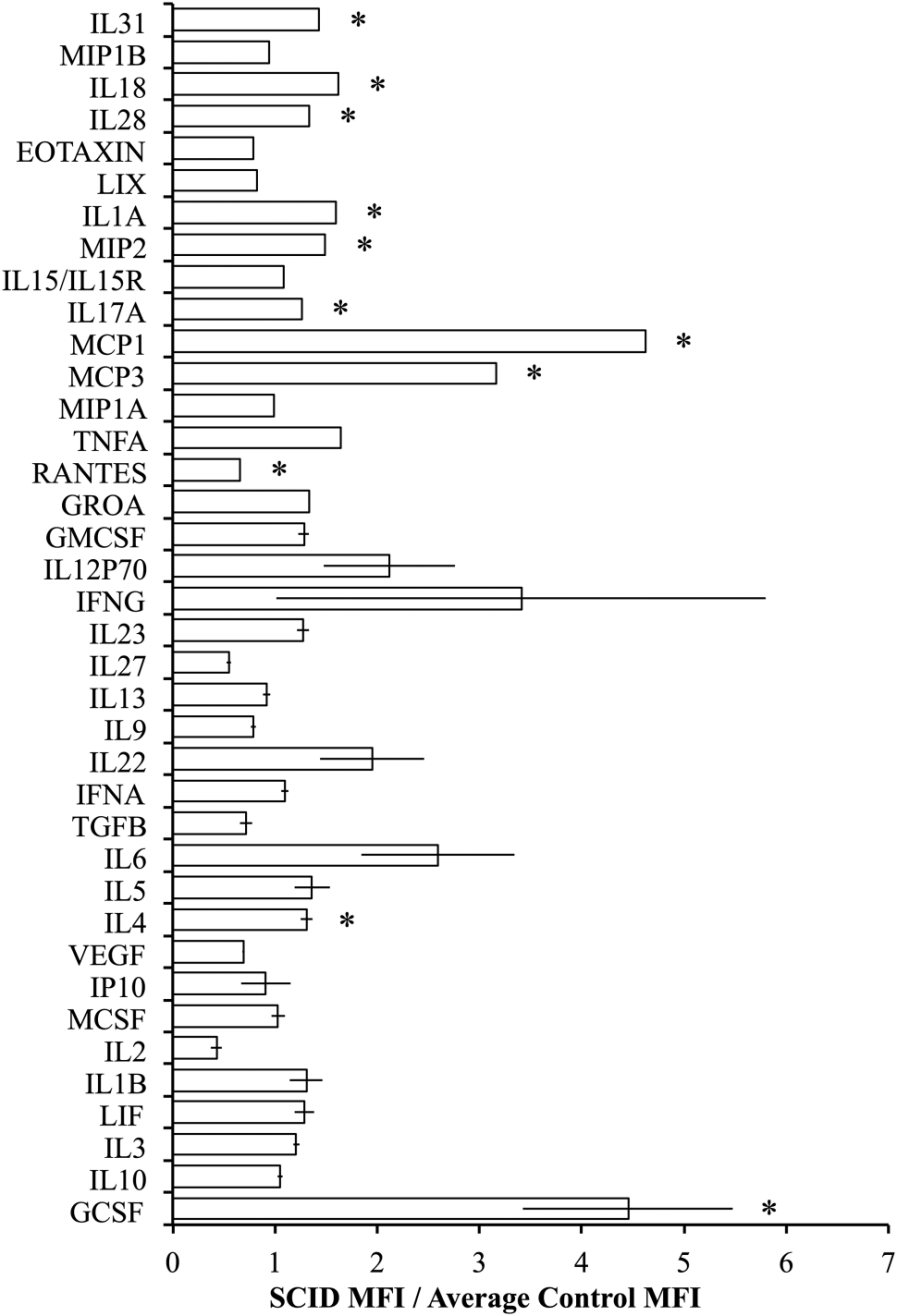
BALB/c *scid* mice have higher levels of macrophage-stimulating cytokines in circulation compared with BALB/c control mice. The average fold difference in the (MFI) of SCID mice relative to the average MFI of control mice (n=5). (Error bars represent the mean±SEM, and *signifies a significant difference between the control and SCID groups of p<0.05.) GCSF, granulocyte colony-stimulating factor; GMCSF, granulocyte macrophage colony-stimulating factor; GROA, growth-regulated protein alpha; IFNA, interferon alpha; IFNG interferon gamma; IL, interleukin; IP, interferon-inducible protein-10; LIX, lipopolysaccharide-inducible CXC chemokine; MCSF, macrophage colony-stimulating factor; MFI, median fluorescence intensity; MCP, monocyte chemoattractant protein; MIP, macrophage inflammatory protein; TGFB, transforming growth factor beta; TNFA, tumor necrosis factor alpha; VEGF, vascular endothelial growth factor.

Luminex was used to evaluate the circulating cytokine profiles of control and SCID mice and revealed that SCID mice had alterations in the levels of circulating cytokines with significantly higher levels of granulocyte colony-stimulating factor (GCSF), IL-4, monocyte chemoattractant protein 3 (MCP3), MCP1, IL-17A, macrophage inflammatory protein 2 (MIP2), IL-1A, IL-28, IL-18, and IL-31 and significantly lower levels of C-C motif chemokine ligand 5 (CCL5) (RANTES) relative to controls (figure 3). The cytokines with the greatest difference in SCID mice were MCP1 and MCP3 and GCSF which were both significantly higher in the immunodeficient SCID mice (p=0.0116, 0.0011, and 0.0207, respectively).

**Figure 4:**
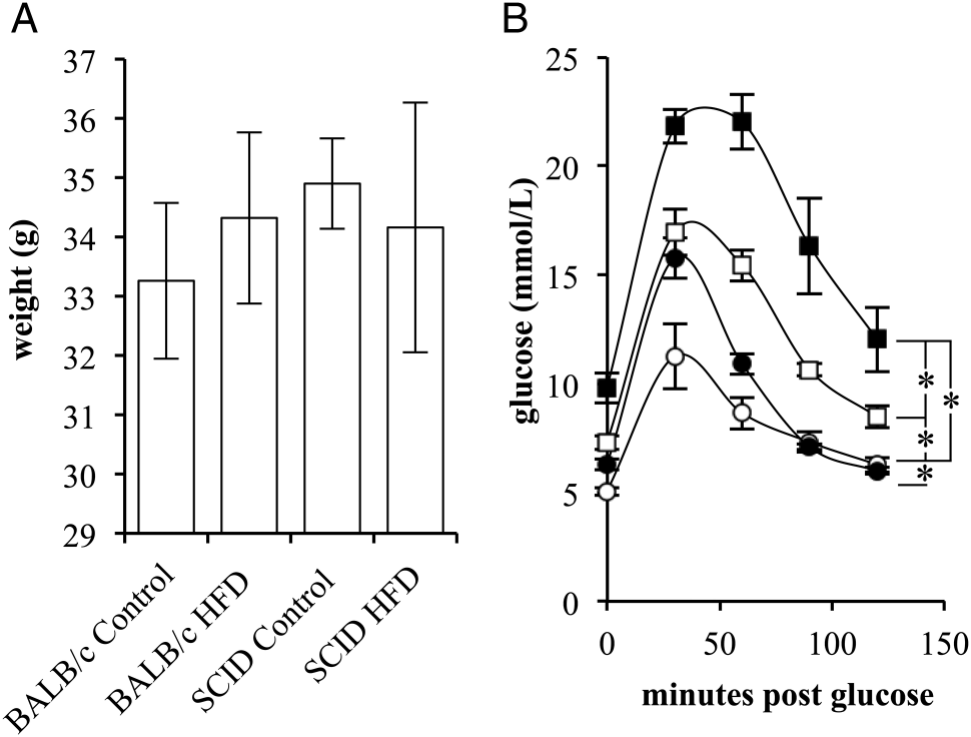
High-fat diet induces progression of glucose intolerance in BALB/c *scid* mice. (A) The total body weight of control (BALB/c) and BALB/c *scid* (SCID) mice following 14 weeks of a control diet (control) or high-fat diet (HFD; n=5). (B) Glucose tolerance test of BALB/c (circle) and SCID (square) mice on the control (white) or HFD (black) diet following a 2 g/kg glucose challenge (n=5). (Error bars represent the mean±SEM, *signifies a significant difference between the designated groups of p<0.05.)

To assess the role of the adaptive immune response in diet-induced glucose intolerance, control (BALB/c) or BALB/c *scid* (SCID) mice were fed either a HFD, with 60% of the kilocalories derived from fat, or a control diet, with 10% of the kilocalories derived from fat, for 14 weeks. At completion of the dietary intervention, there was no significant difference between the total body weights for all groups of mice (figure 4A). BALB/c mice fed on a HFD had significantly (p=0.0473) reduced glucose tolerance relative to BALB/c mice maintained on a control diet with significantly higher fasting blood glucose levels. The SCID mice maintained on the control diet also had significantly (p=0.0013) greater fasting blood glucose levels relative to the BALB/c mice on the control diet. Similarly, the SCID mice on the control diet had a significantly (p=0.0005) reduced glucose tolerance compared with BALB/c mice on the control diet. SCID mice maintained on a HFD had significantly (p<0.0104) higher fasting blood glucose levels compared with all other mouse groups. Additionally, SCID mice on a HFD had significantly higher blood glucose levels following a glucose challenge when compared with the SCID mice on a control diet (p=0.0027) and the BALB/c mice on a HFD (p=0.0001; figure 4B).

## Discussion

The goal of this work was to assess the role of the adaptive immune system in glucose homeostasis versus diet-induced glucose intolerance. We approached this by comparing the phenotypes of the immunodeficient SCID mouse and its immunocompetent BALB/c strain control. Collectively, our studies indicate that an adaptive immune response is a necessary, physiological component of the metabolic organ required for glucose homeostasis, but its absence does not protect against diet-induced glucose intolerance.

SCID mice were insulin resistant in the absence of obesity and were, in fact, leaner than their counterparts. This result supports the role of the adaptive immune system in metabolic homeostasis by suggesting that obesity is not a required component for immune-mediated disruption in insulin tolerance. The finding that the adaptive immune system is important for glucose homeostasis is consistent with work that demonstrated an early populating of the visceral adipose tissue of lean mice by T regulatory cells in an antigen-dependent fashion. The T regulatory cells performed both inflammatory and metabolic functions.^20^ A recent human trial suggests that a HFD can induce insulin resistance without causing concurrent detectable changes in immune function. While short-term overfeeding did result in insulin resistance, it did not cause significant alternations in the populations of immune cells or cytokine gene expression profiles within the subcutaneous adipose tissue.^23^ These findings support that glucose sensitivity is an extraordinarily complex process, melding both physiological and pathological mechanisms of a multitude of organ systems.

Interestingly, cytokine profile analyses in immuocompetent controls and immunodeficient SCID mice demonstrated several significant differences; however, those cytokines that were elevated to a degree as to be functionally significant were all associated with neutrophil proliferation, GCSF, or macrophage recruitment, MCP1 and MCP3. This is of special note as macrophage accumulation within the adipose tissue, especially visceral adipose tissue, is a consistent finding among insulin-resistant mice and humans and is thought to be a necessary component in diet-induced insulin resistance.^1–3^ Additionally, visceral adipose tissue-associated T cells are thought to be the major source of macrophage-recruiting chemokines.^13^ Our results indicated that the MCP1 and MCP3 levels were elevated in the absence of T cells, suggesting an alternative source for these chemokines. The likely source for these chemokines is adipocytes. Others have demonstrated that adipocytes secrete macrophage-recruiting chemokines and that their expression increases in adipocytes on introduction of a HFD.^24^ ^25^ Collectively, these results support the hypothesis that one role of the adaptive immune system in glucose homeostasis is maintaining levels of macrophage-stimulating cytokines and preventing disruption in glucose homeostasis. Additionally, our work indicates that the absence of functional B and T cells does not provide protection against HFD-induced insulin resistance as SCID mice fed on a HFD, although already insulin resistant, developed even greater glucose intolerance.

Surprisingly, mice fed on a HFD did not gain significant amounts of weight. This result may be due to our low sample size, and/or our use of the BALB/c mouse strain. The BALB/c strain has previously been shown to demonstrate less pathology in association with a HFD when compared with other mouse strains.^26^ An additional finding was that 6-week-old SCID mice on a control diet did not have significantly greater fasting blood glucose levels when compared with same-aged BALB/c mice on a control diet (figure 1A and online supplementary material 1); however, 20-week-old SCID mice on a control diet did have significantly higher fasting blood glucose levels when compared with BALB/c mice on a control diet (figure 4B). This result suggests that the glucose intolerant phenotype of SCID mice worsens with age. It is possible that B and T cells have a developmental and/or continuous regulatory role in adipose biology, influencing the gut microbiome, adipose tissue biology, and the adipose tissue inflammatory tone in a way that is exacerbated by age.^7–21^ ^27^

Collectively, this work supports that the adaptive immune system is part of the metabolic organ system and that disruptions in its function can result in insulin resistance; however, an absence of B and T cells does not protect from diet-induced insulin resistance. These findings emphasise that results collected in immune-manipulated and diet-manipulated mice must be interpreted carefully as they can be due to a disruption in physiological glucose homeostasis, pathological insulin resistance or a combination of these two events.
